# Cortical Brain Injury Causes Retrograde Degeneration of Afferent Basal Forebrain Cholinergic Neurons via the p75NTR

**DOI:** 10.1523/ENEURO.0067-23.2023

**Published:** 2023-08-28

**Authors:** Srestha Dasgupta, Laura E. Montroull, Mansi A. Pandya, Juan P. Zanin, Wei Wang, Zhuhao Wu, Wilma J. Friedman

**Affiliations:** 1 Department of Biological Sciences, Rutgers University, Newark, New Jersey 07102; 2 Helen and Robert Appel Alzheimer's Disease Research Institute, Feil Family Brain and Mind Research Institute, Weill Cornell Medicine, New York, New York 10021

**Keywords:** axon degeneration, basal forebrain neurons, p75NTR, proneurotrophins, TBI

## Abstract

Traumatic brain injury (TBI) elicits neuronal loss at the site of injury and progressive neuronal loss in the penumbra. However, the consequences of TBI on afferent neurons projecting to the injured tissue from distal locations is unknown. Basal forebrain cholinergic neurons (BFCNs) extend long projections to multiple brain regions including the cortex, regulate many cognitive functions, and are compromised in numerous neurodegenerative disorders. To determine the consequence of cortical injury on these afferent neurons, we used the fluid percussion injury model of traumatic brain injury and assessed the effects on BFCN survival and axon integrity in male and female mice. Survival or death of BF neurons can be regulated by neurotrophins or proneurotrophins, respectively. The injury elicited an induction of proNGF and proBDNF in the cortex and a loss of BFCNs ipsilateral to the injury compared with sham uninjured mice. The p75NTR knock-out mice did not show loss of BFCN neurons, indicating a retrograde degenerative effect of the cortical injury on the afferent BFCNs mediated through p75NTR. In contrast, locus ceruleus neurons, which also project throughout the cortex, were unaffected by the injury, suggesting specificity in retrograde degeneration after cortical TBI. Proneurotrophins (proNTs) provided directly to basal forebrain axons in microfluidic cultures triggered retrograde axonal degeneration and cell death, which did not occur in the absence of p75NTR. This study shows that after traumatic brain injury, proNTs induced in the injured cortex promote BFCN axonal degeneration and retrograde neuron loss through p75NTR.

## Significance Statement

TBI is well known to elicit direct neuronal loss at the site of injury and secondary loss in the penumbra; however, the effect on afferent neuronal populations that project axons from distal locations such as the basal forebrain has not been elucidated. BFCNs project to a myriad of brain regions and regulate cognitive processes such as learning, attention, and memory, and are compromised in neurodegenerative diseases such as Alzheimer’s disease. These neurons constitutively express p75NTR, a receptor that can promote neuronal degeneration following injury. We demonstrate here that cortical injury promotes degeneration of afferent BFCNs, mediated by p75NTR, indicating that TBI causes neuronal loss in brain regions distal to the site of injury via retrograde axonal degeneration.

## Introduction

Traumatic brain injuries (TBIs) have immediate as well as long-term neurologic consequences and thus progressively affect behavior and quality of life over time. Primary and secondary degeneration as a consequence of TBI has been studied in detail with respect to effects on the injured region and the surrounding penumbra (Loane and Faden, 2010; Montroull et al., 2020; Raghupathi et al., 2000). Secondary injury after cortical TBI includes neuronal, glial, and white matter loss (Raghupathi et al., 2000). Studies using the fluid percussion injury (FPI) model of TBI show a massive change in the injury microenvironment, with an acute inflammatory response, excitotoxicity, increase in reactive oxygen species, as well as changes in neurotrophin mRNA and protein expression (Schimmel et al., 2017; Thompson et al., 2005), which may affect not only the cells in the penumbra but also neuronal populations that extend their axons to cortical targets from distal locations in the brain. Basal forebrain cholinergic neurons (BFCNs), through long and extensive axonal projections, release acetylcholine in the cortex to regulate cognitive functions such as emotion, attention, and memory (Boskovic et al., 2019). They are composed of several nuclei, among which the nucleus basalis of Meynert (NBM) and substantia innominate (SI), comprising the Ch4 cluster, innervate the cortex (Mesulam et al., 1983; Rye et al., 1984). BFCNs require neurotrophic factors for their survival, differentiation, maintenance, and function (Nonomura et al., 1995) that are produced by their neuronal target regions and signal via cognate receptor complexes through the projecting axon terminals. BFCNs are unique in their expression of all the neurotrophin receptors, including the pan-neurotrophin receptor p75NTR as well as the receptor tyrosine kinases TrkA, TrkB, and TkC throughout life. Mature NTs promote a prosurvival response through Trk signaling (Alderson et al., 1990; Hefti et al., 1985; Friedman et al., 1993; Kromer, 1987), whereas proneurotrophins (proNTs) bind p75NTR and sortilin to promote apoptosis in BFCNs (Volosin et al., 2006). The degenerative role of p75NTR in BFCNs has been established in mass cultures and after seizure conditions *in vivo* (Volosin et al., 2006). Proneurotrophins, which are high-affinity ligands for p75NTR, are upregulated in the region of injury (Alder et al., 2016; Sebastiani et al., 2015), and they promote apoptotic signaling in the injury penumbra in the cortex via p75NTR (Montroull et al., 2020), which is a known contributor to secondary neurodegeneration after TBI (Delbary-Gossart et al., 2016; Alder et al., 2016; Montroull et al., 2020). However, whether the changes in the neurotrophic environment of BFCN terminals after TBI have a retrograde effect on survival of afferent projections has not been investigated. The constitutive expression of p75NTR in BFCN throughout life, coupled with the complexity of maintaining an elaborate axonal arbor, may make BFCNs vulnerable to degeneration. Previous studies suggest that TBI increases the risk of neurologic disorders such as Alzheimer’s disease (Tajiri et al., 2013), and BFCN loss is a hallmark of this disease (Whitehouse et al., 1982, 1983).

In our study, moderate cortical FPI was used to investigate the retrograde effect of cortical injury on the projecting BFCNs. Retrograde degeneration through p75NTR has been studied in peripheral neurons (Sørensen et al., 2003; Yano et al., 2009) but not in the context of brain injury and its effect on afferent neurons that project to the injury site from distal locations. We investigated the effect of cortical injury on the afferent BFCN neurons and compared p75NTR knock-out (KO) mice with wild type (WT) mice to understand the role of p75NTR in retrograde BFCN degeneration after injury. To specifically determine the effects of proNTs on retrograde axonal degeneration of basal forebrain neurons, neurons were cultured in microfluidic chambers to investigate whether direct stimulation of axon terminals with proneurotrophins can signal through p75NTR to affect BFCN axon integrity and cell death. These findings indicate that the constitutive expression of p75NTR in BFCNs promotes cell-type-specific retrograde degeneration of BFCNs following cortical TBI.

## Materials and Methods

### Reagents

Recombinant human proNGF (cleavage resistant) protein (catalog #N-285) and recombinant mouse proBDNF (cleavage resistant) protein (catalog #B-243) were purchased from Alomone Labs. Poly-d-lysine, glucose, transferrin, insulin, putrescine, selenium, progesterone, penicillin, and streptomycin were purchased from Sigma-Aldrich. Minimum Essential Medium (MEM), Ham’s F-12 Media, and B-27 Plus Supplement (50×; catalog #A3582801) were purchased from Invitrogen. Microfluidic chambers were prepared using microfluidic chamber master molds, a gift from Eran Perlson (Tel Aviv University), using the protocol described by Harris et al. (2007). Cholera Toxin Subunit B (CTB; recombinant), Alexa Fluor 488 conjugate (catalog #C34775) was purchased from Invitrogen. Propidium iodide (PI; catalog #P1304MP) was obtained from Invitrogen. Cytoplasmic dynein inhibitor Ciliobrevin D (catalog #250410) was purchased from Calbiochem. Antibody to BDNF (catalog #327-100; RRID:AB_2927780) was obtained from Icosagen. Anti-NGF (catalog #N6655; RRID:AB_477660) and mouse anti-β-actin (catalog #A5441; RRID:AB_476744) antibodies was purchased from Sigma-Aldrich. Goat anti-choline acetyltransferase (ChAT; catalog #AB144-P; RRID:AB_2079751), and rabbit-anti-p75NTR (catalog #07-476; RRID:AB_310649) were purchased from Millipore. Goat-anti-p75NTR antibody (catalog #AF1157; RRID:AB_2298561) was purchased from R&D Systems. Mouse-anti-tyrosine hydroxylase (TH; catalog #58844S; RRID:AB_2744555) was obtained from Cell Signaling Technology. Mouse anti-β-III Tubulin (Tuj1; catalog #G712A; RRID:AB_430874) antibody was purchased from Promega. Alexa Fluor 488 (catalog #A-11055) and Alexa Fluor 555 (catalog #A-31572) anti-goat and anti-rabbit secondary antibodies, respectively, and Alexa Fluor 647 anti-mouse secondary antibody (catalog #A32787) were purchased from Invitrogen. Donkey anti-goat Alexa Fluor 647 (catalog #705-607-003; RRID:AB_2340439) was obtained from Jackson ImmunoResearch. Mouse 800 (catalog #926-32210, RRID:AB_621842), rabbit 800 (catalog #926-32213, RRID:AB_621848), and mouse 680 (catalog #926-68020, RRID:AB_10706161) secondary antibodies for Western blots were purchased from LI-COR Biosciences. Fast Blue (5%) was purchased from Polysciences (catalog #73819-41-7). DRAQ5 (catalog #DR05500) was obtained from Biostatus. Fluoromount-G (catalog #0100-01) and DAPI Fluoromount-G (catalog #0100-20) were obtained from Southern Biotech.

### Mice

All experiments were performed in compliance with the Institutional Animal Care and Use Committee policies and approved by Rutgers University. Adult mice between the ages of 2 and 3 months were maintained on a 12 h light/dark cycle with *ad libitum* access to food and water. WT mice were purchased from The Jackson Laboratory; *p75NTR* global KO mice with an exon III deletion (Lee et al., 1992) are bred in house. Both males and females were used in all experiments.

### Neuronal cultures

WT and *p75NTR* KO pregnant mice were killed by exposure to CO_2_ and soaked in 70% ethanol for 5 min for sterilization. Embryonic day (E)15 mouse fetuses were removed under sterile conditions and kept in PBS on ice. Basal forebrains were dissected and dissociated in serum-free medium (SFM; Friedman et al., 1993) composed of a 1:1 mixture of Eagle's MEM and Ham's F-12 supplemented with glucose (6 mg/ml), putrescine (60 μm), progesterone (20 nm), transferrin (100 μg/ml), selenium (30 nm), penicillin (0.5 U/ml), and streptomycin (0.5 μg/ml). The cells were then plated in microfluidic chambers (Taylor et al., 2005) attached to glass coverslips in tissue culture dishes that were precoated overnight with poly-d-lysine (0.2 mg/ml). Then 200 μl of media were added to the soma compartment, and 100 μl media were maintained in the axon compartments to maintain a media volume difference that facilitated the growth of axons through the microgrooves toward the distal compartment. The cells were maintained with the volume difference between the soma and axon compartments in SFM supplemented with 1% B-27 for 5 d at 37°C to obtain compartmentalized BFCN cultures that could be treated separately at the axons or somas.

### Live imaging of BFCNs in microfluidic cultures

BFCN microfluidic cultures were prepared for live imaging after 5 d *in vitro* (DIV). The axon compartment was treated with Alexa 488 labeled CTB (1 μg/ml), a retrograde tracer, for 20 min and washed twice with SFM plus 1% B27 to retrogradely label the BFCNs, which extended axons to the distal compartment through the microgrooves. After 5 h, the CTB from the axons was found to be transported into the cytoplasm of the BFCNs that projected axons to the distal compartment. The soma compartment was treated with PI (1 μg/ml) to label dying neurons. BFCNs were then treated with proNGF (20 ng/ml) or proBDNF (40 ng/ml) in the axon compartment and compared with control untreated compartmentalized BFCNs to assess the effect of axonal stimulation with proNTs on neuronal degeneration. Growth media volume difference was maintained as described in the neuronal culture method to restrict stimulation with proNTs exclusively to the axons. To assess surviving versus dying neurons, live imaging of neurons was performed using a Zeiss LSM 510 confocal microscope maintaining constant temperature (37°) and CO_2_ (5%) for the duration of the experiment. Incorporation of PI in the nucleus of CTB-positive neurons after 24 h axonal treatment was assessed.

### Lateral FPI

TBI was induced in mice using the FPI model following a protocol adapted from Alder et al. (2011) Adult mice (3–5 months of age) were anesthetized with ketamine (80 mg/kg) and xylazine (10 mg/kg). Craniotomy was performed on the right cortical hemisphere midway between bregma and lambda, 2 mm lateral to the midline, 3 mm in diameter. One day after craniotomy we performed a moderate FPI using 30 psi or 2 atm injury pressure. Sham mice underwent craniotomy but were not subjected to FPI. Sham and injured mice were injected with buprenorphine (0.05 mg/kg weight) after the craniotomy and the injury. Injured and sham mice were perfused 1–14 d postinjury (DPI) to obtain brain sections for immunohistochemistry or killed by CO_2_ exposure to obtain brain lysates for analysis by Western blot.

### Western blot analysis of TBI mouse brains

Sham and TBI mice were killed 1DPI, 3DPI, or 7DPI by exposure to CO_2._ Brains were dissected on ice to obtain the area of craniotomy in cortex, as well as the basal forebrain tissue from the injured and uninjured hemisphere, and lysed in 300 μl of RIPA lysis buffer containing the following: NP40 (10%), deoxycholic acid (10%), SDS (10%), EDTA (0.5 m), NaCl (5 m), Tris (1 m), and protease and phosphatase inhibitors. After protein quantification, equal amounts of protein were run on a 15% polyacrylamide gel and transferred to nitrocellulose membrane. Equal protein loading was assessed by Ponceau staining, which was washed out with TBS with 0.05% Tween 20 (TBST). The membranes were then blocked with 5% nonfat milk prepared in TBST for 1 h and incubated with primary antibodies to BDNF (catalog #327-100, Icosagen; RRID:AB_2927780) or NGF (catalog #N6655, Sigma-Aldrich; RRID:AB_477660) overnight. After washing 3 × 10 min with TBST, the blots were incubated with appropriate secondary antibodies for 1 h at room temperature. The membrane was washed 3 × 10 min with TBST and then scanned with the Odyssey infrared imaging system (LI-COR Biosciences). The same procedure was repeated with antibodies to β-actin (catalog #A5441, Sigma-Aldrich; RRID:AB_476744). LC tissue was harvested from naive WT and p75KO mice and processed for quantifying the expression of p75NTR using rabbit anti-p75NTR (1:1000; catalog #07-476, Millipore; RRID:AB_310649), TH using mouse anti-TH (1:1000; catalog #58844S, Cell Signaling Technology; RRID:AB_2744555), and β-actin.

### All blots shown are representative of at least three independent experiments

#### Immunocytochemistry

Basal forebrain microfluidic cultures were fixed with 4% paraformaldehyde for 20 min, washed with PBS, and permeabilized with 0.5% Triton X-100 in PBS for 10 min. The cells were then blocked for 1 h with 5% normal goat serum and 1% bovine serum albumin (BSA) in PBS and incubated overnight at 4°C with primary antibody prepared in 1% BSA in PBS. Primary antisera were directed against Tuj1 (mouse, 1:1000; catalog #G712A, Promega; RRID:AB_430874), rabbit anti-p75NTR (1:1000; catalog #07-476, Millipore; RRID:AB_310649), goat anti-ChAT (1:1000, catalog #AB144-P, Millipore; RRID:AB_2079751). Cells were then washed with PBS, exposed to the appropriate secondary antibodies coupled to different fluorophores, and highly cross-adsorbed against different species (Alexa 488, Alexa 594, and Alexa 647; Invitrogen). Coverslips were mounted on slides using DAPI Fluoromount-G to label the nuclei. Images were obtained using a Zeiss LSM 510 META confocal microscope and analyzed to measure axon fragmentation using ImageJ software. The degeneration index was calculated as the ratio of the area of fragmented axons over the total area of axons (intact axons plus fragmented axons) by using Tuj1-stained fluorescence images. A total of eight images were analyzed per chamber. All images were processed using ImageJ software. To analyze size fragment of particles, binary masks were created of each image. Particles with a size area equal to or lower than 60 µm^2^ and with a circularity index higher than 0.03 were classified as degenerated neurite fragments.

#### Immunohistochemistry

TBI and sham animals were anesthetized with ketamine/xylazine 7DPI and 14DPI and perfused with PBS followed by 4% paraformaldehyde. After perfusion, the brains were removed and postfixed in 4% paraformaldehyde overnight and cryoprotected in 30% sucrose for 2 d; 20 μm sections were obtained using a cryostat (Leica) and mounted onto charged slides and stored at −20°C. The basal forebrain was analyzed by staining coronal sections starting from bregma 0.50 mm to bregma 0.50 mm, with an interval of 200 μm between each section spanning the diagonal band of Broca (DBB), NBM and SI, which comprise the Ch4 nuclei of the basal forebrain (Mesulam et al., 1983). To analyze the locus ceruleus, coronal brain sections (20 μm) starting from bregma −5.00 mm to bregma −6.0 mm, with an interval of 200 μm between each section were processed. Sections were processed as above and then exposed overnight at 4°C to the following primary antibodies: rabbit anti-p75NTR (1:1000; catalog #07-476, Millipore; RRID:AB_310649), goat anti-ChAT (1:1000; catalog #AB144-P, Millipore; RRID:AB_2079751), mouse anti-TH (1:1000; catalog #58844S, Cell Signalling Technology; RRID:AB_2744555) diluted in 1% BSA in PBS. The next day slides were washed three times in PBS for 10 min each and exposed for 1 h at room temperature to secondary antibodies coupled to the Alexa 488 or 555 fluorophores (1:1000) prepared in 1% BSA in PBS. Sections were then washed again in PBS three times for 10 min each. Sections were coverslipped with DAPI Fluoromount-G and analyzed by fluorescence microscopy using a Nikon Eclipse microscope. ChAT- and p75NTR-labeled neurons in the basal forebrain and TH-labeled neurons in the locus ceruleus were counted using ImageJ software.

#### Fast Blue injection and retrograde tracing in vivo

Following craniotomy on the right cortical hemisphere midway between bregma and lambda, 2 mm lateral to the midline, 3 mm in diameter, adult WT mice were injected with Fast Blue (0.25%; catalog #73819-41-7, Polysciences) in five injection sites at two layers in the cortex in the center of the craniotomy, and four injections 1.5 mm from the center diametrically opposite each other, 90° apart. Injections were performed at a rate of 35 nl/min, 30 nl per injection site, at depths of 300 µm and 450 µm, targeting cortical layers 4 and 5, which receive projections from the nucleus basalis and substantia innominate of the basal forebrain, with a 5 min wait period between each injection. Injected brains were harvested 14 d after injection. Sections of the basal forebrain and LC were obtained as described for the immunohistochemistry and processed by immunostaining for rabbit anti-p75NTR (1:1000; catalog #07-476, Millipore; RRID:AB_310649), goat anti-ChAT (1:1000; catalog #AB144-P, Millipore; RRID:AB_2079751), mouse anti-TH (1:1000; catalog #58844S, Cell Signaling Technology; RRID:AB_2744555) and DRAQ5 (1:1000; catalog #DR05500, Biostatus). Sections were coverslipped with Fluoromount-G, and fast-blue-positive cells were analyzed by fluorescence microscopy using Nikon Eclipse microscope and processed using ImageJ software. 

#### Whole-mount imaging

Fixed adult mouse brains with unilateral FPI treatment to induce TBI were delipidated with a modified Adipo-Clear protocol (Hou et al., 2021). Briefly, perfusion-fixed brain samples were washed with B1n buffer (H_2_O/0.1% Triton X-100/0.3 m glycine, pH 7), then transferred to a methanol gradient series (20, 40, 60, 80%) in B1n buffer, 4 ml for each brain, 1 h for each step; then 100% methanol for 1 h; then overnight incubation in a 2:1 mixture of DCM/methanol and 1.5 h incubation in 100% DCM the following day; then 100% methanol for 1 h three times and reverse methanol gradient series (80, 60, 40, 20%) in B1n buffer, 30 min for each step. Samples were then washed in B1n buffer for 1 h and overnight. The above procedures were conducted at room temperature with rocking to complete delipidation. The delipidated samples were then blocked in PTxwH buffer (PBS/0.1% Triton X-100/0.05% Tween 20) with 5% DMSO and 0.3 m glycine for 3 h and overnight at 37°C, then washed with PTxwH for 1 h, 2 h, and overnight at room temperature. For staining, brain samples were incubated in primary antibody (goat anti-p75NTR, 1:500; catalog #AF1157, R&D Systems; RRID:AB_2298561) diluted in PTxwH for 14 d at 37°C. After primary antibody incubation, samples were washed in PTxwH for 1 h, 2 h, 4 h, overnight, then 1 d three times, and then incubated in secondary antibody (Alexa Fluor 647 donkey anti-goat, 1:100; catalog #705-607-003, Jackson ImmunoResearch) diluted in PTxwH for 10 d. Samples were then washed in PTxwH for 1 h, 2 h, 4 h, overnight, then 1 d three times. Samples were finally washed in PBS for one d then proceeded for clearing with iDISCO+ (Hou et al., 2021). Samples were dehydrated with methanol gradient with water, then 100% methanol, DCM/methanol mixture overnight, and 100% DCM for 1 h twice the next day. Brains were finally cleared for 4 h in dibenzyl ether and then stored in a fresh tube of dibenzyl ether before imaging with a LifeCanvas SmartSPIM Light Sheet Microscope. A 647 nm laser was used for whole-mount immunohistochemistry imaging with the 3.6×/0.2 detection lens. Light sheet illumination is focused with NA 0.2 lens and axially scanned with electrically tunable lens coupled to the camera (Hamamatsu Orca-Fusion BT) in slit mode. Camera was set at fast mode (2ms exposure) with 16-bit image format. The *X*/*Y* sampling rate was 1.866 μm and *Z* step at 2 μm. Three-dimensional imaging datasets were processed using ImageJ software, with max-intensity-projection function to generate flattened views of the selected brain volumes in coronal direction.

#### Experimental design and statistical analyses

Statistical analysis was performed using Prism 5.0 software (GraphPad), and image analysis was performed using ImageJ software. All measurements are shown as mean ± SEM. For samples defined by one factor, data were analyzed by one-way ANOVA with Tukey's *post hoc* multiple-comparison test when three or more independent group of samples were compared. For samples defined by two factors, data were compared by two-way ANOVA with Sidak’s *post hoc* multiple-comparison test. For *in vivo* experiments, sample size (*n*) was defined as the number of mice that were quantified. For the *in vitro* experiments, sample size (*n*) was defined as the number of independent cultures of embryos obtained from separate pregnant rats. The null hypothesis was rejected at the 0.05 level; *p* values < 0.05 are considered significant. The statistical test, sample size (*n*), and the *p* values are reported in the figure legends specific to each experiment. Epifluorescent images were assembled using Adobe Photoshop.

## Results

### Cortical FPI causes an induction of proneurotrophins in the cortex ipsilateral to the injury

**Figure 1.**
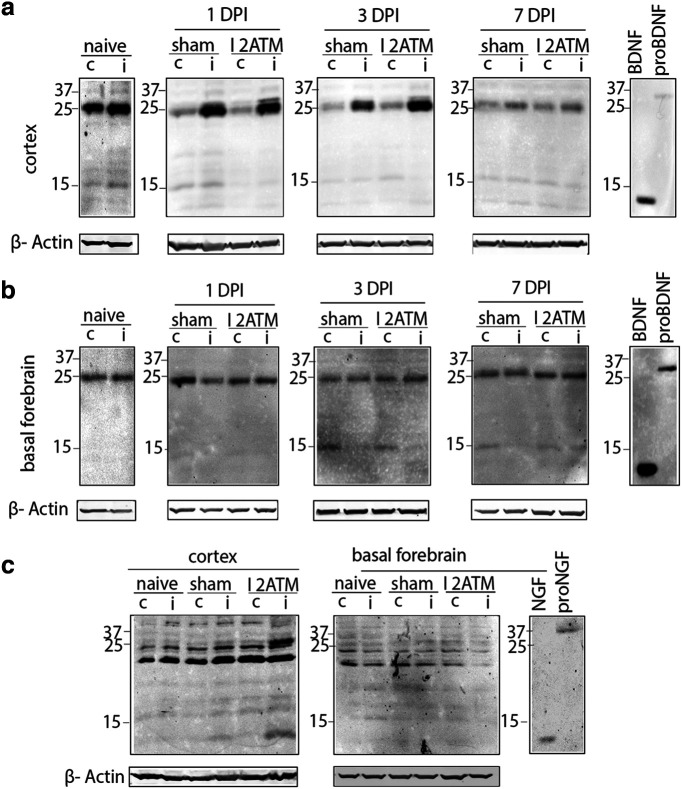
Proneurotrophins are induced in the ipsilateral cortex but not the basal forebrain after cortical FPI. ***a–c***, Brain tissue lysates from naive, sham, and injured (2 atm) wild-type adult mice were obtained 1DPI, 3DPI, and 7DPI to determine levels of proBDNF (***a***, ***b***) and proNGF (***c***) in the injured versus uninjured side. Cortical tissue lysate (***a***) harvested for Western blot was probed for proBDNF (32 kDa) in the ipsilateral and contralateral cortex at 1DPI, 3DPI, and 7DPI in naive, sham, and injured mice. Basal forebrain tissue lysate (***b***) harvested for Western blot was probed for proBDNF (32 kDa) in the ipsilateral versus contralateral basal forebrain at 1DPI, 3DPI, and 7DPI in naive, sham, and injured mice. Cortex and basal forebrain tissue lysates (***c***) harvested for Western blot were probed for proNGF (37 kDa) at 3DPI after FPI in the ipsilateral versus contralateral side of the cortex and the basal forebrain; *n* = 4 (naive), *n* = 4 (sham 1DPI), *n* = 4 (injured 1DPI), *n* = 4 (sham 3DPI), *n* = 4 (injured 3DPI), *n* = 3 (sham 7DPI), *n* = 3 (injured 7DPI; ***a***, ***b***); *n* = 3 (naive), *n* = 4 (sham 3DPI), *n* = 4 (injured 3DPI; ***c***). The established size of proBDNF is 32 kDa; however, a prominent band of 25 kDa was also recognized by the BDNF antibody that appeared to be regulated by injury, but the identity of that band is unclear.

To investigate the effect of moderate cortical injury on afferent basal forebrain neurons, we first examined proNT induction at the region of injury in the cortex as well as in the basal forebrain. Cortical FPI was performed on WT adult mice, and tissue lysates were collected 1DPI, 3DPI, and 7DPI from both hemispheres of the naive, sham, and TBI mouse brains from the cortex and basal forebrain for biochemical analysis of proNTs. A dramatic increase in proBDNF was observed at the injury site of the cortex ipsilateral to the injury in comparison with the contralateral side at 1DPI and 3DPI, which was reduced by 7DPI in injured mice (Fig. 1*a*). Naive mice without a craniotomy or injury had comparable levels of proBDNF (Fig. 1*a*) and proNGF (Fig. 1*c*) in the right and left hemisphere of the cortex and basal forebrain. A trend toward proBDNF induction was observed in sham mice because of the craniotomy, but no difference was observed in comparison with the contralateral side of the cortex (Fig. 1*a*). No changes in proBDNF levels were observed in the basal forebrain at 1DPI, 3DPI or 7DPI (Fig. 1*b*). In addition to proBDNF, a clear induction of proNGF was observed at the injury site in the cortex compared with the contralateral side at 3DPI (Fig. 1*c*), but no changes in proNGF levels were observed in the basal forebrain (Fig. 1*c*). These results indicate that after moderate cortical FPI proneurotrophins are induced in the injured cortex in target brain regions of the basal forebrain neurons but not locally near the basal forebrain soma.

### Cortical FPI promotes a retrograde loss of afferent basal forebrain neurons ipsilateral to the injury

**Figure 2.**
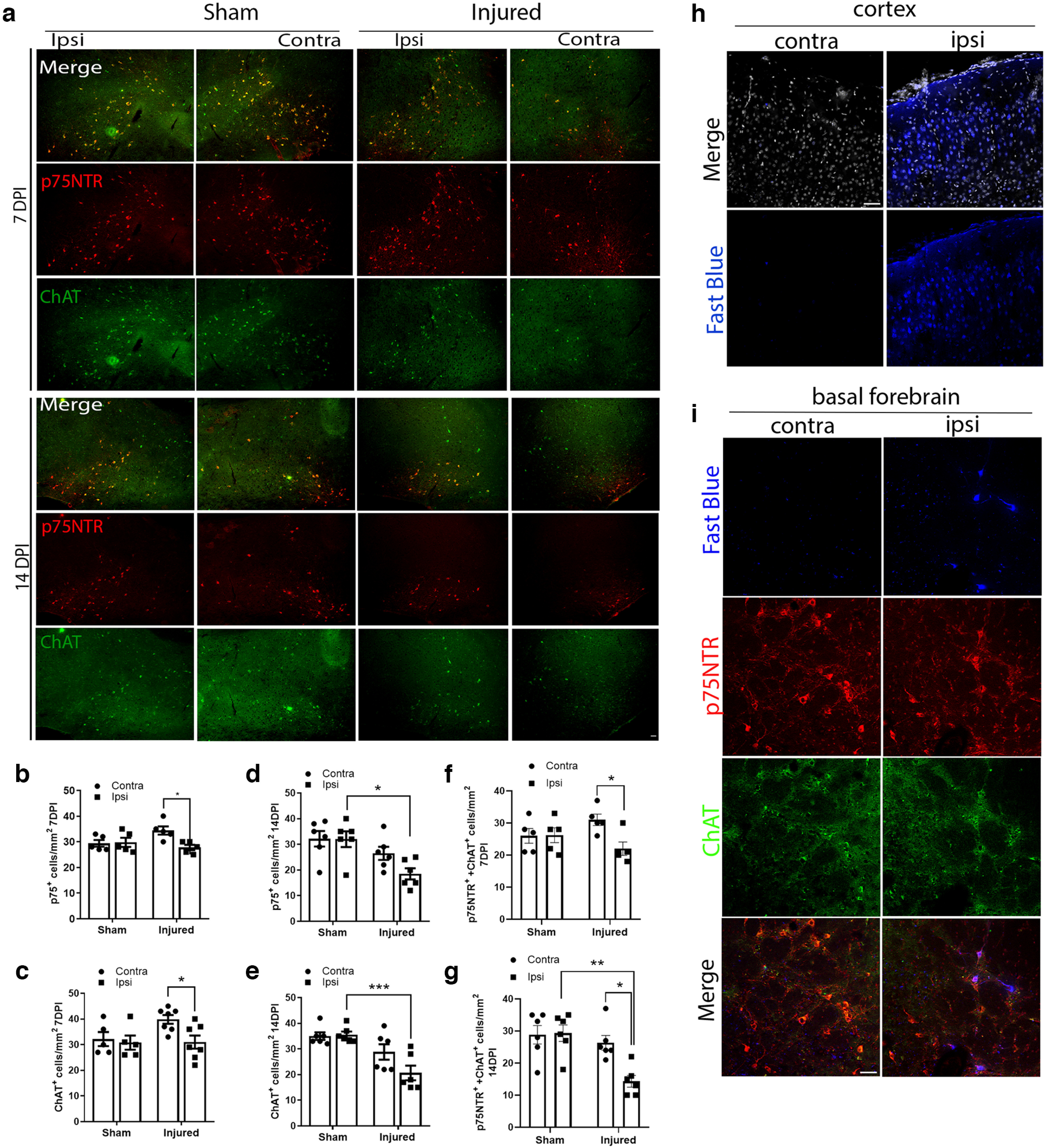
Cortical FPI leads to a retrograde loss of afferent basal forebrain neurons ipsilateral to the injury 7DPI and 14DPI. ***a***, Coronal brain sections of the basal forebrain show immunostaining for p75NTR (red) and ChAT (green) 7 and 14 d after injury in sham and injured mice. Scale bar, 50 μm. ***b***, Quantification of p75NTR+ basal forebrain neurons ipsilateral (Ipsi) to the injury in comparison with the contralateral (Contra) side in sham and injured mice at 7DPI; *n* = 5 sham and 5 injured mice, 7DPI; *p = 0.0257 comparing contra versus ipsi in injured mice. Sham contra versus injured contra, n.s., *p = 0.1121. ***c***, Quantification of ChAT+ basal forebrain neurons ipsilateral to the injury in comparison with the contralateral side in sham and injured mice at 7DPI; *n* = 6 sham and 7 injured mice, *p = 0.0487 comparing contra versus ipsi in injured mice. Sham contra versus injured contra, n.s., *p = 0.1521. ***d***, Quantification of p75NTR+ basal forebrain neurons ipsilateral to the injury in comparison with the sham mice at 14DPI; *n* = 6 sham and 6 injured mice, 14DPI, *p = 0.0109 comparing sham ipsi versus injured ipsi. Injured contra versus injured ipsi, n.s., *p = 0.1937. ***e***, Quantification of ChAT+ basal forebrain neurons ipsilateral to the injury in comparison with sham mice at 14DPI; *n* = 6 sham and 6 injured mice, 14DPI; *p = 0.0010 comparing sham ipsi versus injured ipsi. Injured contra versus injured ipsi, n.s., *p = 0.0883. ***f***, Quantification of p75NTR and ChAT double-positive basal forebrain neurons ipsilateral to the injury in comparison with the contralateral side in sham and injured mice at 7DPI; *n* = 5 sham and 5 injured mice, 7DPI; *p = 0.0391 comparing contra versus ipsi in injured mice. Sham contra versus injured contra, n.s., *p* = 0.3753. ***g***, Quantification of p75NTR and ChAT double-positive basal forebrain neurons ipsilateral to the injury in comparison with the contralateral side in sham and injured mice at 14DPI; *n* = 6 sham and 6 injured mice, 14DPI, *p = 0.0112 comparing contra versus ipsi in injured mice; *p* = 0.0016 comparing sham ipsi versus injured ipsi. Statistical analysis was performed by two-way ANOVA, Sidak’s multiple-comparison tests. ***h***, Coronal brain sections of the naive cortex showing immunostaining for Fast Blue (blue) and DRAQ5 (gray) 14 d after cortical Fast Blue injection in the uninjected versus injected side; *n* = 4. Scale bar, 50 μm. ***i***, Coronal brain sections of the naive basal forebrain showing immunostaining for Fast Blue (blue), p75NTR (red), and ChAT(green) 14 d after cortical Fast Blue injection in the uninjected versus injected side. Scale bar, 50 μm.

Studies reporting neuronal loss in the acute as well as chronic phases after TBI have been limited to the cortical penumbra of the injury, attributed in part to the concurrent induction of proNTs and increased expression of their cognate receptor p75NTR in the injured cortex (Alder et al., 2016; Montroull et al., 2020). However, the effect of cortical FPI on afferent neurons that project to the injured area from distal locations has not been explored. WT naive, sham, and injured mice were analyzed for the number of surviving neurons expressing p75NTR and ChAT, well-established markers for the basal forebrain cholinergic population, throughout the diagonal band of Broca, nucleus basalis, and substantia innominate (Fig. 2*a*). Cortical FPI induced a significant reduction in p75NTR+ neurons after 7 d in comparison with the contralateral side and in comparison with sham uninjured animals (Fig. 2*a*,*b*). A greater effect on p75NTR+ neuron loss was observed after 14DPI (Fig. 2 *a*,*d*). A significant loss of ChAT+ neurons (Fig. 2*a*) was also observed in the basal forebrain 7DPI (Fig. 2*c*) and 14DPI (Fig. 2*e*). Although a trend toward an increase in p75NTR and ChAT+ BFCNs in the contralateral side was observed after 7 d postinjury, the trend was not found to be significant (p75NTR+ Sham contra vs injured contra, n.s., *p* = 0.1121; ChAT+ Sham contra vs injured contra, n.s., *p* = 0.1521). Coimmunolabeling with Ki67, a proliferation marker and p75NTR did not show any proliferating cells in the injured versus uninjured basal forebrain (data not shown). A significant loss of ipsilateral p75NTR and ChAT double-positive neurons was observed compared with the contralateral side in injured mice, which was absent in sham mice (Fig. 2*f*,*g*). Although a trend toward reduction in p75NTR and Chat-positive BFCNs was observed in the contralateral side 14DPI, the effect was not significant compared with the contralateral side in sham mice (Sham/Contra vs Injured/Contra, *p* = 0.8846). Retrograde tracing using Fast Blue injections in the cortex showed Fast Blue+ cells in the cortex and in the basal forebrain that coexpress p75NTR and ChAT on the ipsilateral side of the injection (Fig. 2*h*,*i*). However, no fast-blue-positive cells were observed on the contralateral basal forebrain, suggesting the absence of contralateral connections from the basal forebrain to the injection site in the cortex (Fig. 2*i*). These results suggest that BFCNs undergo retrograde cell death after cortical FPI, which leads to a progressive loss of p75NTR+ and ChAT+ BFCNs 7 and 14 d after injury.

### Cortical FPI does not promote neuronal loss of afferent locus ceruleus neurons ipsilateral to the injury

**Figure 3.**
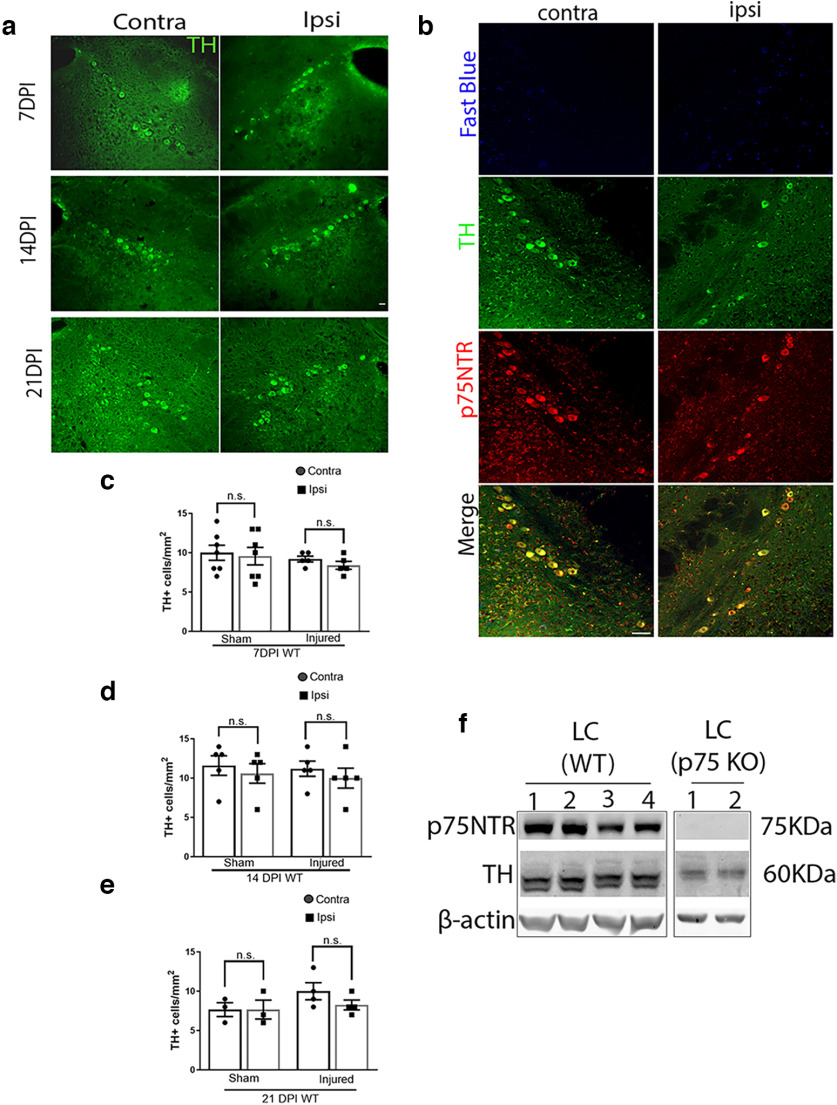
TH-positive cells in the locus ceruleus after cortical FPI; 7DPI, 14DPI, and 21 DPI sham and TB1 brains were obtained from wild-type adult mice after FPI. ***a***, Coronal brain sections of the LC immunostained for TH (green) 7DPI, 14DPI, and 21DPI. ***b***, Coronal brain sections of the naive LC showing absence of staining for Fast Blue (blue) 14 d after cortical Fast Blue injection in the uninjected versus injected side, coimmunolabeled with TH (green) and p75NTR (red); *n* = 4. ***c***, Quantification of TH+ neurons in the ipsilateral versus contralateral side of the LC 7DPI in sham and injured mice. ***d***, Quantification of TH+ neurons in the ipsilateral versus contralateral side of the LC 14DPI in sham and injured mice. ***e***, Quantification of TH+ neurons in the ipsilateral versus contralateral side of the LC 21DPI in sham and injured mice. Statistical analysis was performed using one-way ANOVA, Tukey’s multiple-comparisons test. ***f***, Western blot showing expression of p75NTR and TH in the LC in naive WT mice, along with a negative control using LC from p75NTR KO mice. Each Western blot lane represents an *n*; *n* = 5 WT LC, *n* = 4 p75NTR KO LC. Scale bars: 50 μm.

To compare the effects of FPI on different afferent neuronal populations, we investigated whether cortical FPI promotes a similar degenerative effect on LC afferent neurons as on the BFCNs. The LC noradrenergic neurons send long axonal projections throughout the cortex (Jones and Moore, 1977; Nomura et al., 2014; Pickel et al., 1974). Brain sections through the LC were examined to quantify the number of TH+ neurons in sham and injured WT mouse brains (Fig. 3). No loss of TH+ neurons was observed in the LC ipsilateral or contralateral to the injury at 7DPI (Fig. 3*a*,*c*), 14DPI (Fig. 3*a*,*d*) or even after 21DPI (Fig. 3*a*,*e*), in contrast to the loss of basal forebrain neurons ipsilateral to the injury (Fig. 2), suggesting that cortical FPI does not promote neuronal loss in all afferent neurons. Interestingly, the TH-positive neurons in the LC were found to coexpress p75NTR even in adulthood (Fig. 3*b*). This was also observed by Western blot analysis of LC tissue from WT naive mice, where p75NTR and TH were detected (Fig. 3*f*). However, Fast Blue injections in the craniotomy site at the cortex (Fig. 2*h*), did not result in any fast-blue-positive cells in the ipsilateral or contralateral LC in TH+p75NTR+ neurons even 14 d after Fast Blue injection (Fig. 3*f*), which is in contrast to results observed in the basal forebrain (Fig. 2*i*), suggesting that the LC neurons may not be projecting to the specific cortical injury site. Overall, these results indicate the specificity of retrograde basal forebrain neuronal loss after cortical injury.

### p75NTR is necessary for retrograde loss of afferent basal forebrain neurons after cortical FPI

**Figure 4.**
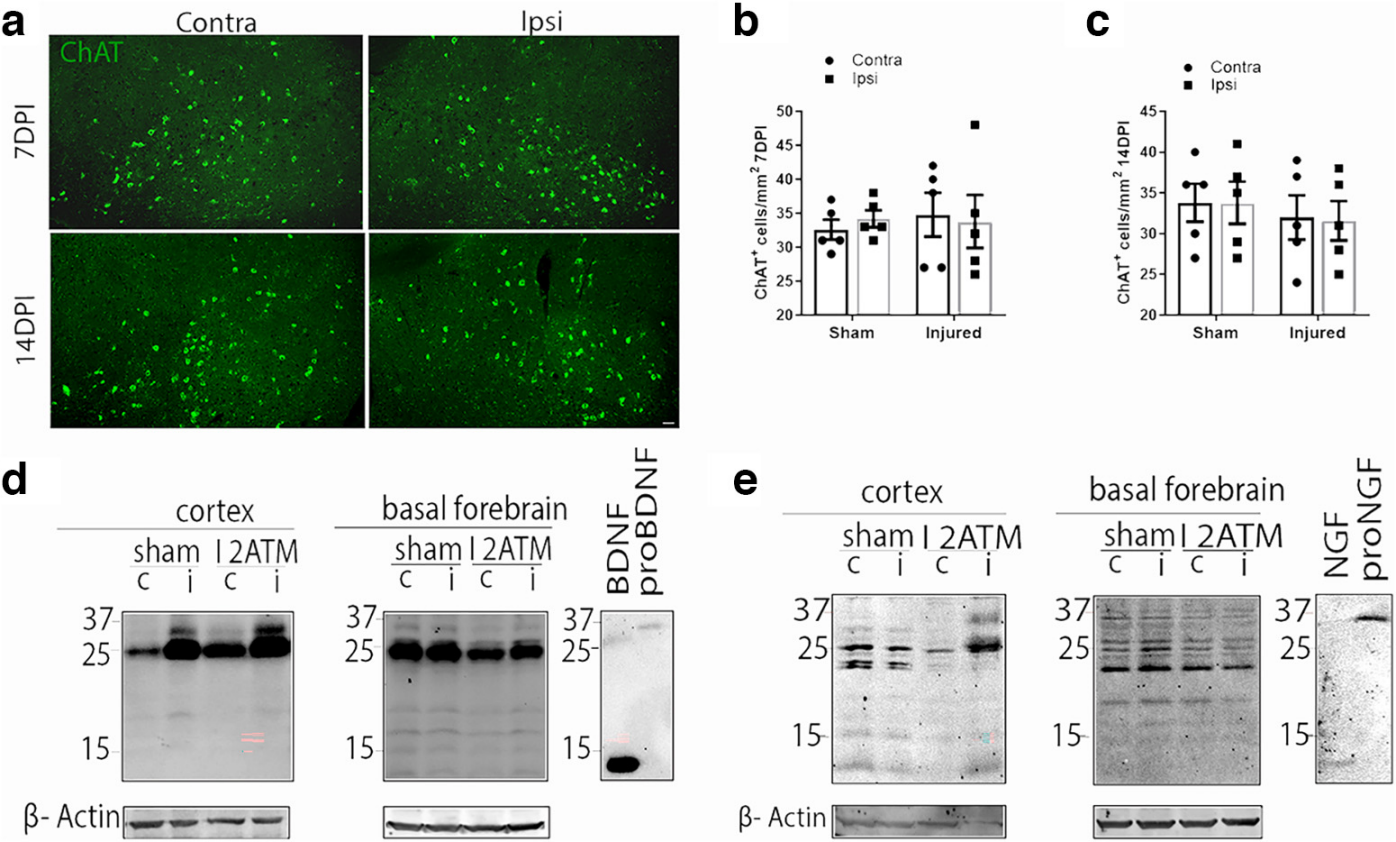
The absence of p75NTR abrogates the retrograde loss of projecting basal forebrain neurons after cortical FPI. ***a***, Coronal brain sections of the basal forebrain from injured *p75NTR* KO mice show immunostaining for ChAT (green) at 7DPI and 14DPI. Scale bar, 50 μm. ***b***, Quantification of ChAT+ BFCNs in the ipsilateral versus contralateral side of the basal forebrain 7DPI in sham and injured p75NTR KO mice. Statistical analysis was performed using two-way ANOVA, Sidak’s multiple-comparisons tests. ***c***, Quantification of ChAT+ BFCNs in the ipsilateral versus contralateral side of the basal forebrain 14DPI in sham and injured p75NTR KO mice. Statistical analysis was performed using two-way ANOVA, Sidak’s multiple comparisons tests. ***d***, Cortex and basal forebrain tissue lysates obtained from 3DPI sham and TB1 *p75NTR* KO mice were probed for proBDNF (32 kDa) by Western blot; *n* = 3 sham and 3 injured brains. ***e***, Cortex and basal forebrain tissue lysates harvested from 3DPI sham and TB1 *p75NTR* KO mice were probed for proNGF (37 kDa) by Western blot; *n* = 3 sham and 3 injured brains.

To investigate the role of p75NTR in BFCN loss after cortical injury, moderate cortical FPI was performed on adult *p75NTR* knock-out mice (*p75NTR* KO; Fig. 4*a*). No significant changes in the number of ChAT+ BFCNs were observed in the absence of p75NTR (Fig. 4*a*) in the ipsilateral versus contralateral basal forebrain of the p75NTR KO mice after cortical FPI 7DPI (Fig. 4*b*) or 14DPI (Fig. 4*c*) in contrast to WT mice (Fig. 2). To assess whether proNTs were still induced after cortical FPI in the *p75KO* mice as observed in WT mice, brain lysates were obtained from *p75NTR* KO naive, injured, and sham mice 3DPI and analyzed by Western blot for levels of proNTs. A dramatic increase in levels of proBDNF and proNGF were detected in the injured cortex in comparison with the uninjured side in the p75NTR KO mice at 3DPI (Fig. 4*d*,*e*), similar to the results observed in WT mice (Fig. 1). No changes in proNT levels were seen in the BF as observed in the WT mice (Fig. 4*d*,*e*). These results show that although FPI induced elevated proNT levels in the *p75KO* mice as in WT mice, no loss of BFCNs occurred in the absence of p75NTR, indicating that retrograde degeneration of BFCNs after FPI requires p75NTR.

### Cortical FPI promotes axonal degeneration of afferent basal forebrain neurons ipsilateral to the injury 7DPI

**Figure 5.**
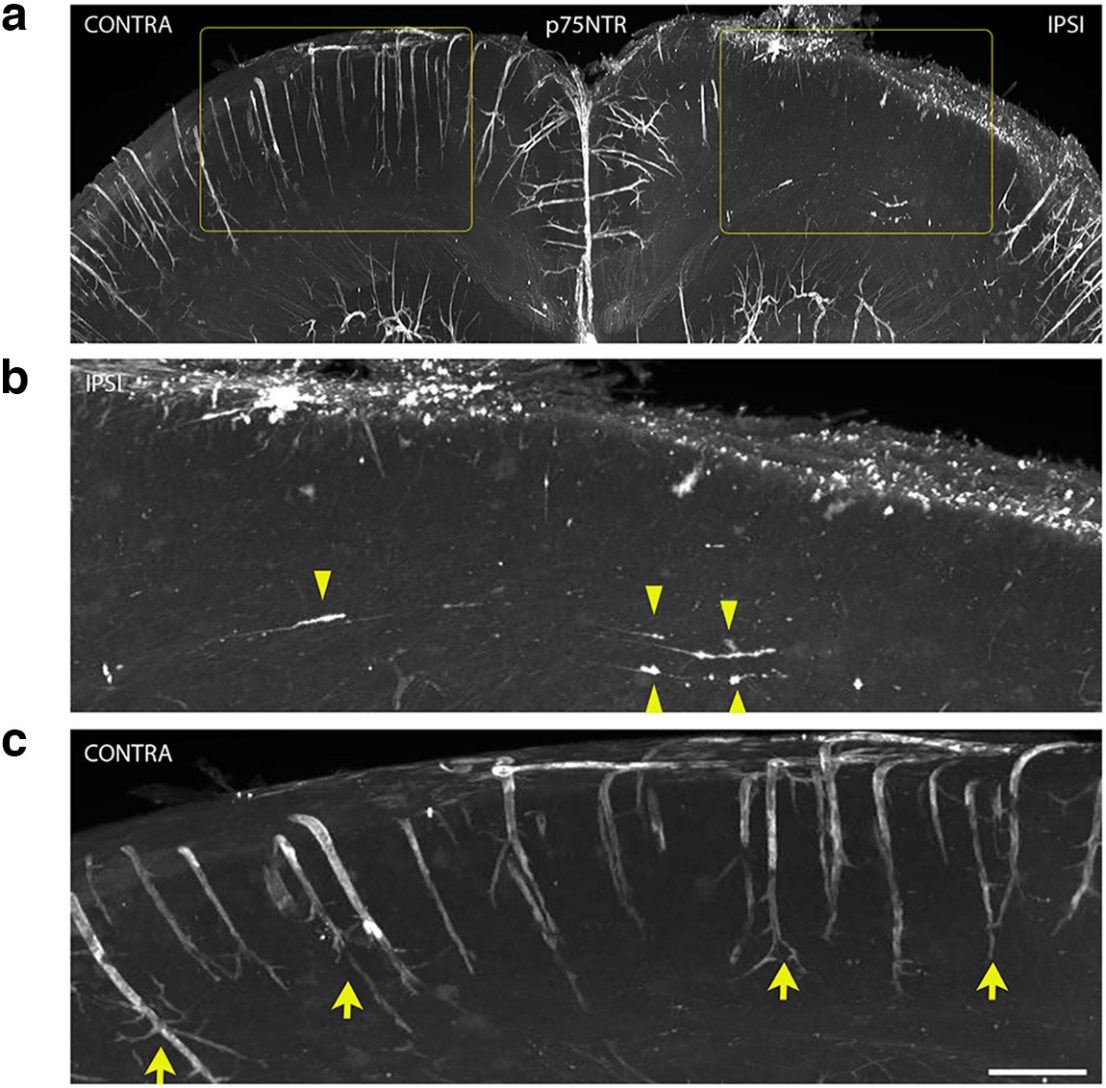
Cortical FPI leads to a retrograde axonal degeneration of afferent basal forebrain neurons ipsilateral to the injury 7DPI. ***a–c***, WT mouse brains were fixed 7DPI after cortical FPIs were cleared by iDISCO before immunostaining for p75NTR. Whole brains were analyzed by light sheet microscopy. Areas highlighted in rectangles (yellow) are magnified (***b***, ***c***) to show the IPSI and CONTRA regions in further detail. p75NTR staining ipsilateral to the injury (***b***) shows p75NTR+ basal forebrain afferents with varicosities, tortuosity, and retraction bulbs (yellow arrowheads). p75NTR staining in the cortex contralateral to the injury (***c***). Yellow arrows denote p75NTR+ blood vessels in the uninjured cortex; *n* = 3 (7DPI). Scale bar, 50 μm.

WT mouse brains with moderate FPI were fixed 7DPI and processed for iDISCO whole-mount immunolabeling with anti-p75NTR to investigate the consequences of cortical FPI on the projecting BFCN axon integrity (Fig. 5*a*). Induction of p75NTR expression at the cortical injury site was observed in accordance with previous studies (Montroull et al., 2020; Fig. 5*a*,*b*). Additionally, the ipsilateral (IPSI) side of the brain showed p75NTR+ axon projections with varicosities, tortuosity, and retraction bulbs extended toward the injured cortex (Fig. 5*b*, yellow arrowheads) indicative of axon degeneration, suggesting that axonal integrity of projecting BFCNs was compromised. In contrast, the uninjured or contralateral (CONTRA) side of the brain was devoid of degenerating p75NTR+ axons (Fig. 5*c*). These results suggest that retrograde degeneration of basal forebrain afferent axons occurs after injury to the cortex, leading to loss of basal forebrain neurons. Blood vessels in the mouse brain express abundant levels of p75NTR, although the function of this receptor in blood vessels is unknown (Fig. 5*c*, yellow arrows). Interestingly, p75NTR expression in the blood vessels was lost in the region of injury (Fig. 5*b*).

### Proneurotrophins signal through p75NTR to promote retrograde degeneration of basal forebrain cholinergic neurons *in vitro*

**Figure 6.**
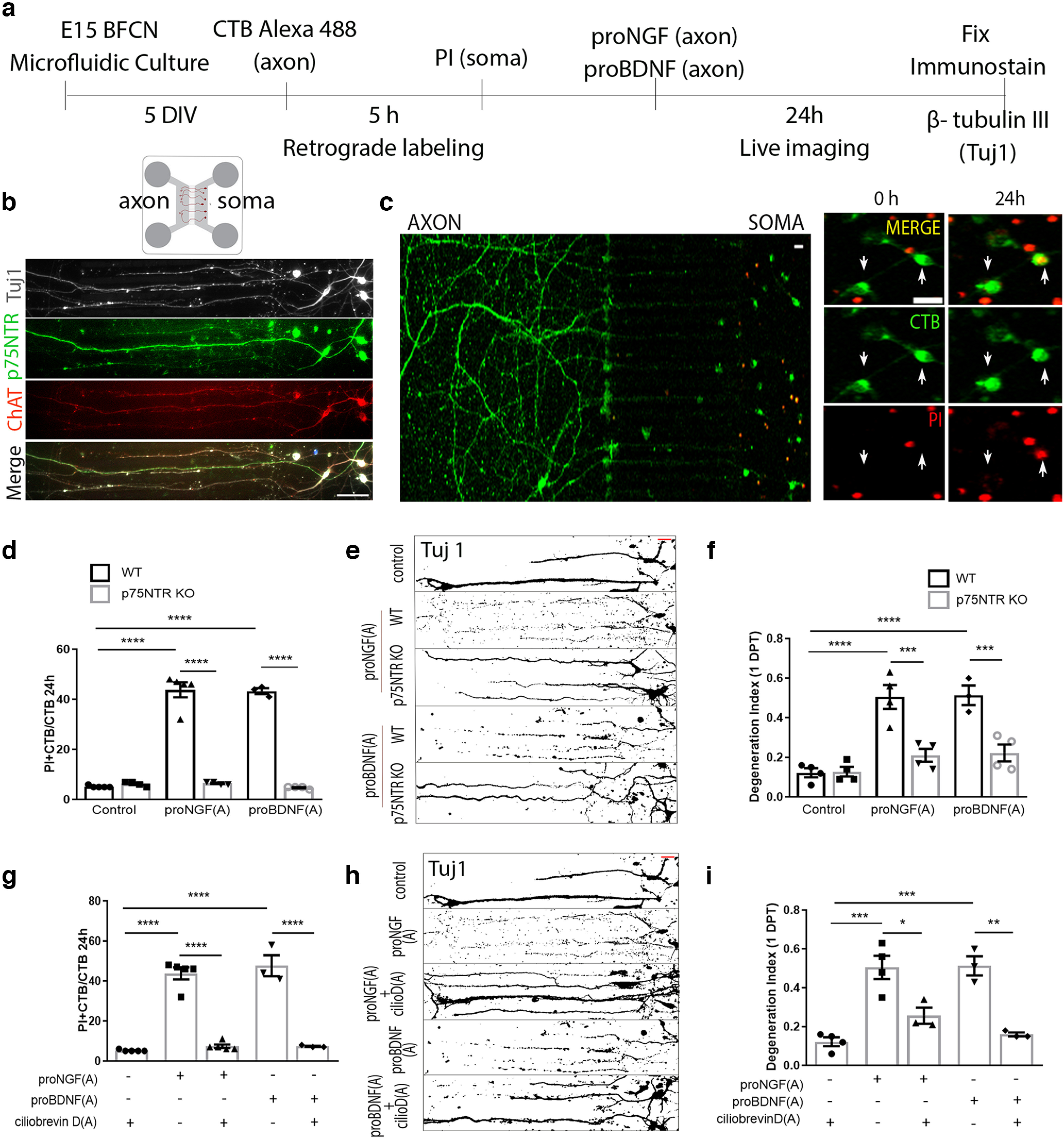
proNGF and proBDNF promote retrograde degeneration of BFCNs in microfluidic cultures via p75NTR. ***a***, Basal forebrain neurons were cultured from E15 mouse embryos in microfluidic chambers for 5DIV. ***b***, BFCNs grown in microfluidic chambers coimmunolabeled for Tuj1 (gray), p75NTR (green), and ChAT (red). Scale bar, 50 μm. ***c***, The axon compartment was treated with CTB Alexa 488 (green) to retrogradely trace neurons that extended their axons to the distal compartment. PI (red) was added to the soma compartment before axonal treatment to study dying (PI+/CTB+) neurons after 24 h of axonal treatment with proneurotrophins. Arrows indicate CTB+ neurons that incorporate PI in their nucleus after 24 h of treatment. Scale bar, 20 μm. ***d***, Quantification of dying neurons in WT and p75NTR KO cultured BFCNs after axonal treatment with proNGF or proBDNF; *n* = 5 (Control, WT), *n* = 4 (Control, KO), *n* = 5 [proNGF(A), WT)], *n* = 4 [proNGF(A), KO], *n* = 3 [proBDNF(A), WT], *n* = 4 [proBDNF(A), KO], where (A) indicates axonal treatment; ****p < 0.0001 by two-way ANOVA, Sidak’s multiple comparisons tests. ***e***, Axon fragmentation in WT or KO BFCNs after proNGF or proBDNF treatment assessed using Tuj1 staining represented as binary images. Scale bar (red, top right), 20 μm. ***f***, Quantification of axonal degeneration in WT and *p75NTR* KO cultured BFCNs after axonal treatment with proNGF or proBDNF; *n* = 4 (Control, WT), *n* = 4 (Control, KO), *n* = 4 [proNGF(A), WT], *n* = 4 [proNGF(A), KO], *n* = 3 [proBDNF(A), WT], *n* = 4 [proBDNF(A), KO]; ****p < 0.0001 comparing WT/control versus proNGF(A), and WT:control versus proBDNF(A), ****p = 0.0007 comparing WT:proNGF(A) versus KO:proNGF(A), ***p = 0.0007, and WT:proBDNF(A) versus KO:proBDNF(A) ***p = 0.0007 by two-way ANOVA, Sidak’s multiple comparisons tests. ***g***, Quantification of dying neurons in WT cultured BFCNs with or without pretreatment with ciliobrevin D (50 μm) before proNGF or proBDNF treatment in the axons; *n* = 5 (Control), *n* = 5 [proNGF(A)], *n* = 5 [proNGF(A)+ CilioD(A), KO], *n* = 3 [proBDNF(A)], *n* = 3 [proBDNF(A)+ CIlioD(A)]; ****p < 0.0001 by one-way ANOVA, Tukey’s multiple comparison tests. ***h***, ***i***, Axon fragmentation in WT cultured BFCNs with or without pretreatment with ciliobrevin D (50 μm) before proNGF or proBDNF treatment in the axons assessed using Tuj1 staining represented as binary images (***h***) and quantification of axonal degeneration (***i***); *n* = 4 (Control), *n* = 4 [proNGF(A)], *n* = 3 [proNGF(A)+ CilioD(A)], *n* = 3 [proBDNF(A)], *n* = 3 [proBDNF(A)+ CilioD(A)]. Scale bar, ***h*** (red, top right), 20 μm. In *i* the asterisk indicates ***p = 0.0001 comparing control versus proNGF(A), ***p = 0.0002 comparing control versus proBDNF(A), *p* = 0.0102 comparing proNGF(A) versus proNGF(A)+ CilioD(A), and **p = 0.0011 comparing proBDNF(A) versus proBDNF(A)+ CilioD(A) by one-way ANOVA, Tukey’s multiple comparison tests.

Studies in mass cultures have demonstrated that proneurotrophins signal through the p75NTR-sortilin receptor complex to promote BFCN death (Volosin et al., 2006). To investigate whether direct stimulation of axon terminals with proneurotrophins can induce retrograde cell death of BFCNs via p75NTR, an *in vitro* microfluidic culture model was used (Fig. 6*b*). BFCNs grown in the microfluidic system extended their axons to the distal compartment over 5DIV and express the BFCN markers ChAT and p75NTR (Fig. 6*b*). Basal forebrain neurons from WT and p75NTR KO mouse embryos were cultured in microfluidic chambers for localized stimulation of the axons (Fig. 6*a*). Axons were treated with Alexa 488-labeled CTB to identify the neurons that projected their axons to the distal chamber. ProNGF or proBDNF was added to the axon compartment, followed by live imaging from 0 to 24 h. PI was added to the soma compartment to monitor dying neurons, and the number of CTB Alexa 488–positive neurons that incorporated PI was quantified in comparison with control untreated BFCNs (Fig. 6*b–d*). Axonal stimulation with proNGF or proBDNF resulted in a significant increase in CTB+/PI+ dying neurons (Fig. 6*c*) quantified as percentage of total CTB+ neurons after 24 h, suggesting that proNGF and proBDNF can promote retrograde cell death initiated from the axons in WT but not p75NTR KO neurons (Fig. 6*d*). To investigate the effect of proNT-p75NTR signaling on axonal integrity, cells were immunostained for Tuj1 after 24 h axonal treatment with proNGF or proBDNF (Fig. 6*e*). Both proNGF and proBDNF treatment of WT BFCN axons for 24 h promoted a significant increase in axon fragmentation in comparison with control untreated WT BFCNs (Fig. 6*e*,*f*). In contrast, proNGF or proBDNF axonal stimulation of p75NTR KO BFCN cultures did not result in BFCN cell death (Fig. 6*d*) nor promote axon degeneration (Fig. 6*e*,*f*). To determine whether retrograde transport was necessary for axonal proNT-induced BFCN degeneration, we inhibited the function of the retrograde motor dynein by pretreating the axon compartment for 20 min with ciliobrevin-D, a dynein functional inhibitor, before axonal stimulation with proNGF or proBDNF. Blocking retrograde motor function significantly rescued the BFCNs from retrograde axon degeneration (Fig. 6*h*,*i*) as well as cell death (Fig. 6*g*) even after 24 h of axonal proNGF or proBDNF stimulation compared with BFCNs that did not receive ciliobrevin-D pretreatment, suggesting that the proNT-p75NTR degenerative signal requires retrograde transport to the soma to promote BFCN axon degeneration as well as cell death. These results demonstrate that proNTs, which are induced in the injured cortex following TBI, can promote retrograde degeneration of afferent basal forebrain neurons via p75NTR, which may contribute to the progressive retrograde loss of these neurons after cortical injury *in vivo*.

## Discussion

Previous studies have shown that brain injury induces increased expression of proneurotrophins and p75NTR at the site of injury and in the penumbra with a prominent role in mediating the secondary neuronal degeneration that occurs in the penumbra after TBI (Alder et al., 2016; Montroull et al., 2020; Sebastiani et al., 2015). The loss of cortical neurons after injury is reduced when p75NTR is deleted or the proNT ligands that bind to this receptor are inhibited (Montroull et al., 2020). However, in addition to the induction of p75NTR on injured neurons in the cortex, this receptor is constitutively expressed on basal forebrain neurons that project their axons throughout the cortex. Therefore, we investigated whether the constitutive expression of p75NTR might render the basal forebrain neurons vulnerable to degeneration because of the induction of proNTs in their cortical target region after TBI, eliciting retrograde cell death initiated at the axon terminal.

### Cortical FPI promotes a retrograde loss of projecting basal forebrain neurons

Proneurotrophin induction is a consequence of TBI in the area of impact (Alder et al., 2016; Montroull et al., 2020), and we confirmed the increase in proBDNF and proNGF by 3 d after the injury. To assess whether there were any consequences for the afferent BFCNs, we examined the number of neurons in the DBB, SI, and NBM that project to the cortex and express p75NTR and ChAT, both well-established markers of basal forebrain cholinergic neurons. Cortical FPI elicited a significant loss of BFCNs that express both p75NTR and ChAT in the basal forebrain ipsilateral to the injury compared with the contralateral side of WT mice, indicating that in addition to the local damage at the site of injury spatially distant neuronal populations such as the BFCNs that send afferent projections to the region of injury may be adversely affected by cortical TBI. The trend toward an increase in contralateral BFCNs observed in injured mice 7DPI was not statistically significant. Retrograde tracing of BFCN afferents that project to the injury site confirmed that BFCNs do not project contralaterally. The trend toward contralateral BFCN loss at 14DPI may be attributed to indirect effects of the injury, such as inflammation. Our previous study had shown that seizure-induced injury in the brain elicited increased proNGF levels in basal forebrain astrocytes with a consequent loss of basal forebrain neurons (Volosin et al., 2006), suggesting that BFCNs may be exposed to altered proNT levels in their local environment after certain types of injury. To assess whether the loss of BFCN was because of increased proneurotrophin expression within the basal forebrain, or alterations in the trophic environment in their injured target regions elicited by TBI, we investigated levels of proNTs in the basal forebrain after injury. Following moderate FPI we found no differences in the basal forebrain between the ipsilateral and contralateral sides of the brain, and no differences compared with sham animals, suggesting that the cortical injury did not induce alterations in proNT levels within the basal forebrain and that the neuronal loss observed in the ipsilateral basal forebrain after FPI may be attributed to the proNT exposure of the BFCN axon terminals at their injured targets.

Traumatic axonal injury in the region of injury has been a long-standing focus of study in relation to secondary degeneration after TBI (Johnson et al., 2013). In addition to progressive neuronal death in the injured cortex as a consequence of TBI (Alder et al., 2016; Montroull et al., 2020), injury-induced axon degeneration in cortical neurons has also been established after frontal TBI (Chen et al., 2009). Using whole-mount immunostaining for p75NTR of injured brains cleared with iDISCO, we identified p75NTR+ axon projections with varicosities, tortuosity, and retraction bulbs extended toward the injured cortex on the ipsilateral side of the brain. These hallmarks of degenerating axons, and their subcortical location, suggest that afferent neurons projecting to the injured cortex undergo retrograde axonal degeneration.

### Specificity of afferent neuronal loss after injury

To assess the specificity of retrograde neuronal loss after TBI, we examined another afferent population of neurons that projects to the cortex, the noradrenergic neurons of the LC.

Interestingly, LC neurons showed no change in the number of TH+ neurons even at 21DPI, suggesting that specific afferent neuronal populations are adversely affected by an injury to their target brain regions, whereas others are spared. Interestingly, the LC neurons were also found to express p75NTR, similar to BFCNs even in adulthood. However retrograde tracing from the craniotomy site in the cortex indicated that the LC neurons may not specifically project to the cortical region targeted for injury in this TBI model and therefore may play a role in the contrasting response noted in the LC compared with the basal forebrain after injury. These observations also suggest that spatial differences in the injury location versus distribution of axonal terminals of projecting neurons determine the degenerative effect on distal neuronal populations. The specificity of the degenerative effect of cortical TBI on BFCNs might also be because of differences in other cell-type-specific protein expression, subcellular localization of components for the required cell signaling cascades, and more, which need further investigation to be elucidated.

To establish whether the loss of BFCN was because of the expression of p75NTR, we compared *p75NTR* KO mice with WT mice. Although proneurotrophins were similarly induced in *p75NTR* KO mice as in WT mice, no loss of BFCNs was seen in the *p75NTR* KO mice after cortical FPI, in contrast to our observations with WT mice, indicating that retrograde neurodegeneration of BFCNs after TBI was mediated by p75NTR.

### Proneurotrophins signal through p75NTR to promote retrograde degeneration of basal forebrain cholinergic neurons *in vitro*

To investigate whether proneurotrophins could directly elicit retrograde degeneration of basal forebrain neurons initiated at the axon terminal, we used *in vitro* microfluidic chambers to separate the axons from the somas. ProNGF or proBDNF treatment of axons elicited axonal fragmentation during 24 h of treatment, leading to neuronal cell death. The mechanisms governing p75NTR-induced apoptosis in cells have been studied in detail in the CNS (Lee et al., 2001; Nykjaer et al., 2004; Troy et al., 2002; Volosin et al., 2006). Previously described downstream signaling mechanisms governing p75NTR-induced cell death, such as the intrinsic caspase pathway (Troy et al., 2002), may be a potential pathway involved in p75NTR-mediated retrograde cell death as well as axon degeneration. However, other established axon degeneration mechanisms (Coleman and Höke, 2020) may also be involved in conjunction with cell death signaling to specifically affect the axonal integrity. Whether the same mechanisms govern p75NTR-mediated axon degeneration and cell death or whether axonal degeneration involves an independent signaling mechanism remains to be investigated.

A major consequence of traumatic brain injury is the progressive neuronal loss that occurs over days and weeks following the initial insult. Previous studies have shown that the induction of p75NTR on injured cortical neurons plays a significant role in mediating neuronal loss in the penumbra of the injury. However, in addition to the local effects of injury eliciting loss of cortical neurons, projecting BFCN afferent neurons that constitutively express p75NTR can respond to proneurotrophins induced by injury to their target and promote retrograde degeneration. Interestingly, an increase in proneurotrophin expression in the cortex has been observed in several conditions of brain insults such as seizures (Friedman, 2010; Volosin et al., 2006) as well as in degenerative diseases such as Alzheimer’s disease (Cuello and Bruno, 2007; Pedraza et al., 2005), which also show BFCN loss. The progressive worsening of cognitive functions such as memory and learning that occurs over time following cortical injuries (Thompson et al., 2005) may be in part because of loss of BFCNs as well as cortical neurons. Rescue of medial septal cholinergic neurons by NGF infusion has been shown to improve cognitive behavior after FPI (Sinson et al., 1997), suggesting that loss of cholinergic basal forebrain neurons contributes to progressive cognitive decline following FPI. The contribution of NBM or SI BFCN loss after TBI remains uninvestigated. Therefore, determining key regulators of the retrograde BFCN degeneration after TBI, as well as parsing out the mechanistic differences between axonal degeneration and cell death signaling in BFCNs after TBI, is essential to our understanding of the spatial impact and temporal aspect of BFCN loss under injury conditions.

## Synthesis

Reviewing Editor: Viji Santhakumar, University of California Riverside Decisions are customarily a result of the Reviewing Editor and the peer reviewers coming together and discussing their recommendations until a consensus is reached. When revisions are invited, a fact-based synthesis statement explaining their decision and outlining what is needed to prepare a revision will be listed below. The following reviewer(s) agreed to reveal their identity: Amber Nolan. Note: If this manuscript was transferred from JNeurosci and a decision was made to accept the manuscript without peer review, a brief statement to this effect will instead be what is listed below.

The study describes a novel injury-induced neurodegeneration in basal forebrain cholinergic neurons (BFCNs) that is mediated by p75NTR. Using the in vivo fluid percussive injury model, the authors show that cortical injury results in proneurotrophin (proNT) upregulation in the cortex concomitant with p75NTR-dependent degeneration of BFCN afferents. Using microfluidic chambers, the authors demonstrate that local proNGF/proBDNF exposure to axons induces p75NTR-dependent axonal and soma degeneration in vitro. Moreover, proNT-induced neurodegeneration required retrograde transport in axons; consistent with the BFCN degenerative event observed in vivo. They show that TH+ neurons in the locus coeruleus do not undergo a similar degeneration. This paper contributes novel insights into the mechanism of retrograde neurodegeneration at the penumbra of traumatic brain injury & establishes proNT-p75NTR signaling as a mediator of this event. There are some statistical concerns and choice of controls that need to be addressed.

1. A major finding in the paper is the loss of P75+ and CHAT+ve neurons in the BFCN. While this is apparent in the 14dpi data, it is not evident in the 7 dpi data. It appears that the ipsilateral, post-injury “neuronal loss” is really an increase in neuronal numbers in the contralateral side after injury. While pairwise comparison is reported, the effect of actors “injury” and “side” are not reported. I suspect there will be an interaction between injury and laterality . Moreover, it looks like counts in contralateral sham vs. injury will be statistically significant but not ipsilateral sham vs injury. Additionally, potential reasons for the apparent increase in P75+ and CHAT+ve neurons in contralateral BF needs to be discussed. Along these line, effect of injury and side need to be presented for the 14 day data as well. Also, at 14 dpi, there almost appears to be a trend towards loss of the contralateral neurons? Does BFCN project contra laterally? It might be helpful to show quantification of neurons expressing BOTH p75NTR and Chat rather than each only alone.

2. In figures 1 and 4, the densitometry and corresponding statistics are not meaningful and need to be removed. The western blots are clear whereas the densitometry causes confusion. To elaborate, proBDNF induction is clear in the ipsilateral 2ATM cortex @ 1dpi & 3dpi. This effect is binary: proBDNF is undetectable in sham & contralateral 2ATM conditions. As a reader, it is hard to trust the densitometry readout of a band that cannot be seen. It’s likely the densitometry signal reflects blot background, rather than a specific readout, in these cases. Moreover, the 2-way ANOVA and Sidak post hoc test is not appropriate here, as the authors set every contralateral band intensity to zero. These zero-error conditions result in artificially low p-values, which can cause misleading conclusions. Rather than run additional blots to fix the statistics, I suggest the authors remove the densitometric analysis altogether - the proBDNF blots are clear and convincing.

3. Figures. In general, it would be helpful to see the actual data points in the bar graphs, and indications of all significant p-values (for example the text states a significant reduction in p75NTR+ neurons at 7 days compared to contralateral AND to sham uninjured animals the later of which isn’t shown on the bar graph). If needed, a table of p-values could be helpful if there are too many to fit on the graphs. Remove the ANOVA p-values in the quantification figures. These should be stated in the figure legend rather than the figure itself.

4. Figure 1, in the text, the authors report proBDNF decrease in the cortex at 7dpi (relative to 3dpi), but not in the basal forebrain, without including the data. Please include the 7 dpi blots. Additionally, Use a different representative proBDNF blo for Fig 1D. There appears to be non-specific background signal in the 1dpi sham contralateral basal forebrain blots.

5. Related to fig. 3, control experiments illustrating p75NTR immunostaining in locus coeruleus sections needs to be included. Please show a side-by-side panels of p75NTR signal in sections of BFCNs vs. LC neurons. While it is expected that the LC neurons will be p75NTR-negative, but this must be confirmed. Also, this could bolster the argument of why BFCNs are sensitive to retrograde neurodegeration in this injury model.

6. Relevant to Figure 6, the authors need to provide control experiments confirming the BFCN identity/proportion of these cells (e.g. ChAT immunostain).

7. The possibility that retrograde axonal degeneration of LC neurons may just take longer due to the location in the midbrain? Do these results hold up 1mo after injury?

8. The findings in figure 2 merit further discussion. proBDNF/proNGF is induced in the cortex at 1-3 dpi, with BFCN neurodegeneration not apparent until 7-14 dpi. Please speak to this temporal lag the Discussion section. The effect is clear, but the reader would like to know your thoughts on why degeneration occurs 4+ days after past cortical proNT induction in vivo.

9. The authors comment on loss of p75NTR signal in blood vessels. This is an interesting observation but co-localization of the p75NTR signal with an appropriate vessel marker is required to draw this conclusion.

10. In figure 5, iDISCO labeling, can the authors quantify the % axons with degenerative properties? Do you see similar axonal profiles closer to basal forebrain nuclei as well? Was co-label for Chat+ axons attempted?

11. In figure 2, representative age-matched ChAT and p75NTR immunostains for brain sections from sham mice need to be included.

12. The fist header in the results section on pg. 12 lists both cortex and hippocampus, yet only cortex is discussed.

13. In results relevant to figure 5, clarify that the loss of p75+ BFCNs was observed at 7dpi

## Author Response

We thank the reviewers for their comments, which are in italics with our responses below.

1. A major finding in the paper is the loss of P75+ and CHAT+ve neurons in the BFCN. While this is apparent in the 14dpi data, it is not evident in the 7 dpi data. It appears that the ipsilateral, post-injury “neuronal loss” is really an increase in neuronal numbers in the contralateral side after injury. While pairwise comparison is reported, the effect of actors “injury” and “side” are not reported. I suspect there will be an interaction between injury and laterality . Moreover, it looks like counts in contralateral sham vs. injury will be statistically significant but not ipsilateral sham vs injury. Additionally, potential reasons for the apparent increase in P75+ and CHAT+ve neurons in contralateral BF needs to be discussed. Along these line, effect of injury and side need to be presented for the 14 day data as well. Also, at 14 dpi, there almost appears to be a trend towards loss of the contralateral neurons? Does BFCN project contra laterally? It might be helpful to show quantification of neurons expressing BOTH p75NTR and Chat rather than each only alone.

• In Fig 2, for 7DPI and 14DPI data we reassessed our statistical data using 2-way Anova, Tukey’s multiple comparisons test, comparing effects of all actors: “sham”, “injured”, “contra” and “ipsi”. Though a trend appears towards an increase in p75NTR+ and ChAT+ BFCNs in the contralateral side at 7 DPI, the trend was not found to be significant (p75NTR+ Sham contra vs injured contra: n.s. p = 0.1121; ChAT+ Sham contra vs injured contra: n.s. p = 0.1521).

• To test for potential reasons for the apparent increase in p75+ ChAT+ neurons in the contralateral BF, co-immunolabeling with Ki67, as a proliferation marker, and p75NTR was done. Our results did not show any Ki67+ cells in the injured or uninjured basal forebrain at 7DPI (data not included in figure, but will be provided if requested), suggesting that there is no increase in neurogenesis in the contralateral basal forebrain after cortical TBI that could lead to the observed trend.

• At 7DPI: p75NTR+ or ChAT+ Contra sham vs Ipsi injured was also found to be not significant. p75NTR+ or ChAT+ Contra sham vs Ipsi sham was also not significant. The only statistically significant effect at 7DPI was found to be within the injured brains between Contra Injured versus Ipsi Injured for both p75NTR and ChAT numbers, indicating a loss of BF neurons on the injured side compared to the uninjured side of the brain.

• At 14DP we found that p75NTR+ or ChAT+ Contra Injured vs Ipsi Injured was not significant (p= 0.1937). This may be due to the observed trend in reduction in p75NTR+ and ChAT+ BFCNs on the contralateral side. However, this reduction in the uninjured side of the brain at 14 DPI was not significant compared to the contralateral side in sham mice (Sham:Contra vs. Injured:Contra: p = 0.8846). Retrograde tracing using Fast blue injections in the location of the craniotomy showed Fast Blue+ cells only in the injected side of the basal forebrain that co-express p75NTR and ChAT (Fig 2. h, i). No Fast blue positive cells were observed in the contralateral basal forebrain, indicating the absence of contralateral connections from the basal forebrain to the injection site in the cortex (Fig. 2 i). The trend towards a reduction of BFCNs in the contralateral side 14DPI may be due to an indirect effect of the TBI over time, possibly due to inflammatory effects of the injury, but not due to direct contralateral connections to the injury area.

• Quantifications of p75 and ChAT double positive neurons have been added (Fig. 2 f, g). At 7DPI, the statistical data of p75+ChAT+ neurons comparing effects of all actors: “sham”, “injured”, “contra” and “ipsi” is similar to the results observed by quantifying p75NTR or ChAT alone. Although the statistical results of 14DPI p75+ChAT+ numbers is similar to 14DPI p75NTR and ChAT alone, a significant effect between “Injury” and “side” was also observed (Contra Injured vs Ipsi Injured: significant. p = 0.0112).

2. In figures 1 and 4, the densitometry and corresponding statistics are not meaningful and need to be removed. The western blots are clear whereas the densitometry causes confusion. To elaborate, proBDNF induction is clear in the ipsilateral 2ATM cortex @ 1dpi & 3dpi. This effect is binary: proBDNF is undetectable in sham & contralateral 2ATM conditions. As a reader, it is hard to trust the densitometry readout of a band that cannot be seen. It’s likely the densitometry signal reflects blot background, rather than a specific readout, in these cases. Moreover, the 2-way ANOVA and Sidak post hoc test is not appropriate here, as the authors set every contralateral band intensity to zero. These zero-error conditions result in artificially low p-values, which can cause misleading conclusions. Rather than run additional blots to fix the statistics, I suggest the authors remove the densitometric analysis altogether - the proBDNF blots are clear and convincing.

• As suggested by the reviewer, the densitometric analysis of blots in Fig 1 and 4 have been removed. The comment that the blots are clear and convincing is much appreciated.

3. Figures. In general, it would be helpful to see the actual data points in the bar graphs, and indications of all significant p-values (for example the text states a significant reduction in p75NTR+ neurons at 7 days compared to contralateral AND to sham uninjured animals the later of which isn’t shown on the bar graph). If needed, a table of p-values could be helpful if there are too many to fit on the graphs. Remove the ANOVA p-values in the quantification figures. These should be stated in the figure legend rather than the figure itself.

• The p-values have been removed from the graphs in all figures as suggested.

• The graphs have been updated to show the actual data points.

• All significant p values are mentioned in the figure legend.

• The text stating “a significant reduction in p75NTR+ neurons at 7 days compared to contralateral AND to sham uninjured animals” may have been misleading. The intent was to express that “a significant reduction in p75NTR+ neurons was observed at 7 days on the ipsilateral side compared to the contralateral side in injured mice, which is not observed in sham mice.”

4. Figure 1, in the text, the authors report proBDNF decrease in the cortex at 7dpi (relative to 3dpi), but not in the basal forebrain, without including the data. Please include the 7 dpi blots. Additionally, Use a different representative proBDNF blo for Fig 1D. There appears to be non-specific background signal in the 1dpi sham contralateral basal forebrain blots.

• 7DPI proBDNF blots for the cortex and basal forebrain have been added to Figure 1a and 1b as requested.

• A different representative blot has been used for proBDNF 1 DPI basal forebrain without non-specific background signal.

5. Related to fig. 3, control experiments illustrating p75NTR immunostaining in locus coeruleus sections needs to be included. Please show a side-by-side panels of p75NTR signal in sections of BFCNs vs. LC neurons. While it is expected that the LC neurons will be p75NTR-negative, but this must be confirmed. Also, this could bolster the argument of why BFCNs are sensitive to retrograde neurodegeration in this injury model.

• Images for p75NTR co-staining with TH in the naïve LC has been included in Fig 3 as suggested. Interestingly, and to our surprise, all TH+ cells in the LC express p75NTR (Fig. 3 b). However, despite the presence of p75NTR, the LC neurons did not show retrograde degeneration in comparison to BFCNs. To investigate whether the LC neurons project specifically to the cortical injury site, sections in the LC obtained from the same Fast blue injected brains discussed for retrograde tracing in the basal forebrain were investigated for Fast blue, p75NTR and TH expression. Fast blue staining was not detected in the LC ipsilateral nor contralateral to the cortical injection site even 2 weeks after the injection, suggesting that the LC neurons might not project directly to the injury site in this region of the cortex. These results suggest a rationale for the lack of retrograde degenerative effect in our TBI model on LC neurons although these neurons express p75NTR.

• The expression of p75NTR was also confirmed using western blot analysis of WT LC tissue, compared with WT basal forebrain tissue as a positive control, and LC tissue from p75NTR KO mice as a negative control (fig. 3 f).

6. Relevant to Figure 6, the authors need to provide control experiments confirming the BFCN identity/proportion of these cells (e.g. ChAT immunostain).

• Image of BFCNs grown in culture co-expressing ChAT, p75NTR, and Tuj1 has been added (Fig. 6 b).

7. The possibility that retrograde axonal degeneration of LC neurons may just take longer due to the location in the midbrain? Do these results hold up 1mo after injury?

• To address the possibility that the LC neurons may just take longer to respond to the cortical TBI, brains were harvested from sham and injured mice as late as 21 DPI and processed for number of TH+ neurons in the ipsilateral versus contralateral side of the LC, but no significant difference was observed (Fig. 3 e). These results, together with the absence of Fast Blue retrograde transport to the LC, suggest that the LC neurons may not project to this cortical region.

8. The findings in figure 2 merit further discussion. proBDNF/proNGF is induced in the cortex at 1-3 dpi, with BFCN neurodegeneration not apparent until 7-14 dpi. Please speak to this temporal lag the Discussion section. The effect is clear, but the reader would like to know your thoughts on why degeneration occurs 4+ days after past cortical proNT induction in vivo.

• The temporal lag in the BFCN loss which is not apparent until 7-14dpi may be attributed to the retrograde signaling mechanisms that underlie the response. The proneurotrophic signal received by the BFCN axon terminals in their cortical targets may potentially activate several signaling cascades which need further elucidation. The time required by the BFCNs to respond to the signal depends on the nature of the retrograde signal which traverses the axon to reach the soma. We observed degenerating BFCN afferents at 7DPI (Fig. 5), which suggests that the process of retrograde degeneration was still ongoing at 7DPI, and the affected BFCNs (and therefore the p75NTR expression) were not already lost. The general timeframe of secondary neurodegeneration that occurs as a response to the primary impact in different TBI models has been reported to be between hours to weeks in the vicinity of the injury and therefore may take longer periods of time to affect distal populations such as the basal forebrain.

9. The authors comment on loss of p75NTR signal in blood vessels. This is an interesting observation but co-localization of the p75NTR signal with an appropriate vessel marker is required to draw this conclusion.

• These observations were drawn using iDISCO labeling of whole brains harvested from injured mice. Detection of blood vessels using endothelial markers in cryosectioned brain tissue may require thicker sections which are unavailable at the moment. Attempts at sourcing the appropriate blood vessel markers are also in process.

10. In figure 5, iDISCO labeling, can the authors quantify the % axons with degenerative properties? Do you see similar axonal profiles closer to basal forebrain nuclei as well? Was co-label for Chat+ axons attempted?

• iDISCO labeling with p75NTR only provides an observable phenotype of the degenerating axons, which are only seen in the ipsilateral side. Retraction bulbs and varicosities of degenerating axons accumulate p75NTR, making them clearly visible, in comparison to intact axons. Therefore, it is very hard to quantify the % axons with degenerative properties. However, the difference between the contralateral and ipsilateral side in this context is very clear. Axonal profiles closer to the basal forebrain nuclei are hard to assess as well due to the density of the staining in the somas.

• iDISCO labeling with ChAT was attempted but unsuccessful.

11. In figure 2, representative age-matched ChAT and p75NTR immunostains for brain sections from sham mice need to be included.

• Representative age matched ChAT and p75NTR immunostains for brains sections from sham mice have been added to Fig. 2 a.

12. The fist header in the results section on pg. 12 lists both cortex and hippocampus, yet only cortex is discussed.

• This was an error, only the cortex has been analyzed.

13. In results relevant to figure 5, clarify that the loss of p75+ BFCNs was observed at 7dpi

• In Fig. 5 clarification has been added that the loss of p75+ BFCNs was observed at 7dpi by mention in the heading.

We hope that these responses are satisfactory to the reviewers and that the manuscript is now acceptable for publication.
